# The protective role of EP300 in monocrotaline-induced pulmonary hypertension

**DOI:** 10.3389/fcvm.2023.1037217

**Published:** 2023-02-22

**Authors:** Lei Yang, Jinglin Tian, Jun Wang, Jie Zeng, Ting Wang, Boya Lin, John Linneman, Li Li, Yanqin Niu, Deming Gou, Yunhui Zhang

**Affiliations:** ^1^Shenzhen Key Laboratory of Microbial Genetic Engineering, Guangdong Provincial Key Laboratory of Regional Immunity and Diseases, Vascular Disease Research Center, College of Life Sciences and Oceanography, Shenzhen University, Shenzhen, Guangdong, China; ^2^Department of Pulmonary and Critical Care Medicine, The First People’s Hospital of Yunnan Province, The Affiliated Hospital of Kunming University of Science and Technology, Kunming, Yunnan, China; ^3^School of Medicine, Washington University in St. Louis, St. Louis, MO, United States

**Keywords:** pulmonary hypertension, EP300, pulmonary arterial smooth muscle cell, cell proliferation, EGR1

## Abstract

**Background:**

Pulmonary hypertension (PH) is a lethal disease characterized by pulmonary vascular remodeling, which is mediated by the abnormal proliferation/migration of pulmonary arterial smooth muscle cells (PASMCs). Recent reports suggest the involvement of histone acetylation in PAH development and that histone deacetylase (HDAC) inhibitors have therapeutic potential for the treatment of PAH. EP300 is an acetyltransferase that plays diverse roles in cell proliferation, differentiation, and apoptosis. However, the functions of EP3000 in PH are rarely studied.

**Results:**

In this work, we found that the expression of EP300 was increased in the pulmonary arteries of monocrotaline (MCT)-induced PH rats. Knockdown of EP300 by AAV-mediated shRNA exacerbated the PH, with a higher right ventricular systolic pressure (RVSP), right ventricular hypertrophy index (RVHI), and wall thickness in the pulmonary artery of MCT-induced PH rat. On the cellular level, the proliferation of PASMCs was promoted by EP300 knockdown. In addition, the expression of EP300 was increased in PASMCs by the overexpression of EGR1, while the deletion of EGR1 binding sites in the EP300 promoter region decreased the activity of EP300 promoter. Moreover, deleting the EP300 promoter region containing EGR1 binding sites using CRISPR/Cas9 abolished the upregulation of EP300 in MCT-induced rats and exacerbated MCT-induced PH. To summarize, our data indicate that EP300 upregulation mediated by EGR1 has a protective effect on MCT-induced PH.

**Conclusion:**

These findings showed EP300 expression was increased in the MCT-induced PH model in rats, which could be mediated by EGR1; the EP300 also displayed the potential to provide protection from PH.

## 1. Introduction

Pulmonary hypertension (PH) is a vascular disease characterized by increased pulmonary arterial pressure and resistance, generally resulting in death from right heart failure (1). Despite the increased understanding of pathology and great advances in therapeutics, PH is still an incurable disease and needs more research work to elucidate its mechanisms. Pulmonary vascular remodeling associated with pulmonary arterial smooth muscle cell (PASMC) hyperproliferation is the essential pathological feature of PH (2). Cytokines, growth factors, environmental conditions, and genetic predispositions are also reported associated with PH. Recently, accumulating evidence suggested that aberrant epigenetic changes, including histone acetylation, also contributed to pulmonary vascular remodeling and PH pathogenesis (3, 4).

Histone lysine acetylation is a fundamental epigenetic mechanism in the regulation of gene expression. Imbalanced histone acetylation could cause abnormal cell functions and further result in complications such as cancer and various cardiovascular diseases (5). The balanced histone acetylation state is dynamically maintained by two families of enzymes: histone acetyltransferases (HATs) and histone deacetylases (HDACs). HATs catalyze the acetylation of lysine residues on core nucleosomal histones, leading to a relaxed chromatin structure and activating transcription. HDACs remove the acetyl groups from histones, leading to chromatin condensation and transcriptional repression (5). Several reports showed that HDACs present abnormal expression in PH patients and animal models (6, 7), suggesting a tight connection between histone acetylation and PH. Moreover, the therapeutic potential of some HDAC inhibitors has been evaluated in rodent models of PH. Zhao et al. (6) reported that both VPA (valproic acid, class I HDACs inhibitor) and SAHA (vorinostat, class I and II HDACs inhibitor) could alleviate hypoxia-induced PH in rats. Two other class I-selective HDAC inhibitors (MGCD0103 and/or MS-275) significantly reduced pulmonary artery systolic pressure in hypoxic-induced rats (8). MC1568, a class II HDACs inhibitor, could also alleviate PH by promoting MEF2 activity (9). The relationship between HDAC action and PH development has been explored extensively. However, whether HAT enzyme participates in PH pathogenesis is rarely studied.

About 30 different HATs have been identified and largely linked to cancer progression (3). PH shares some phenotypes with cancer, however, none of these HATs was evaluated in PH. Among these HATs, EP300 has been investigated extensively and suggested to play a critical role in cardiopulmonary system (10–13), but the elucidation of its role in PH is still scarce (14). EP300 acetylates many non-histone proteins and modulates cell functions *via* regulating stability, cellular localization, and molecular interaction (15). Most of the proteins acetylated by EP300, such as hypoxia-inducible factor 1α (HIF1α), TP53, STAT3, RELA, MYB, NFKB1, FOXO1, MYOD1, and GATA factors (16), have been demonstrated to play crucial roles in PAH (17), implying EP300 might participate in PAH occurrence.

In this study, we found that the interference of EP300 expression promoted PASMCs proliferation and aggravated monocrotaline (MCT)-induced PH in rats. Additionally, EP300 upregulation mediated by EGR1 provided a protective effect for MCT-treated rats.

## 2. Materials and methods

### 2.1. Ethics statement

Sprague–Dawley (SD) rats used in this study were purchased from Guangdong Medical Laboratory Animal Center (Guangzhou, China). All experiments were conducted in accordance with the China Council on Animal Care, and the protocols used were approved by the Animal Care and Use Committee of Shenzhen University.

### 2.2. MCT-induced PH rat model and measurement of hemodynamics

All rats were kept at 22°C with a 12-h light and dark cycle and allowed to feed at will. Male rats (8-week-old) were administrated with MCT (50 mg/kg; Sigma) or an equal volume of saline by subcutaneous injection to induce PH. After 3 weeks, rats were anesthetized by inhalation of isoflurane to measure right ventricle (RV) pressure, and they were placed on a heated table to maintain their temperature during the procedure. The right jugular vein was surgically exposed, and a 2 F Millar Mikro-Tip catheter transducer (Millar Instruments Inc., Houston, TX) was inserted in the RV through the incision in the right jugular vein. The RV systolic pressure (RVSP) was recorded using the MP150 system and AcqKnowledge software package (BIOPAC, Goleta, CA). To assess RV hypertrophy, saline was flushed into the RV after death, and the heart was removed. The RV was separated from the left ventricle (LV) and the ventricular septum (S). The ratio of the weight of the RV and that of the LV plus ventricular septum (LV + S) was used to assess RV hypertrophy.

### 2.3. Isolation of rat pulmonary arteries (PAs)

The experimental tools were disinfected with 75% alcohol in advance and the liquid used is pre-cooled. The anesthetized SD rat was placed on the anatomical board flat on its back, fixed with a tack, peeled away the skin from the lower jaw to the abdomen on both sides, opened the rat’s chest (careful to avoid cutting the internal organs), completely removed the heart and lung tissue, and temporarily immersed in phosphate-buffered saline (PBS). The tissue around the pulmonary artery was observed and cleaned under the anatomic microscope, and the complete pulmonary artery was quickly separated and cleaned in PBS.

### 2.4. Preparation of AAV9 virus and infection in rat lungs

A U6 promoter-driven shRNA expression system, along with CMV-GFP (Green fluorescent protein) cassette used to monitor transduction efficiency was established in a vector of adeno-associated virus 9 (AAV9). The AAV9 vector containing shRNA against EP300 (shEP300) or scramble control sequences (shCon) was co-transfected into 293T cells with two other plasmids (pRC and pHelper). The virus were harvested at 3 days after transfection. The virus titer was detected through absolute quantitative PCR. The resulting AAV9-shP300 titer was determined to be 1.3 × 10^14^ vector genomes (vg)/ml, and the AAV9-shCon titer was 1.5 × 10^14^ vector genomes (vg)/ml. About 100 μL of AAV9 virus (1 × 10^13^ vg/rat) was delivered to the lungs of rats at 7 weeks of age *via* intratracheal injection.

### 2.5. Histological analysis

After fixation, the lung was embedded in paraffin and sectioned (5-μm thickness). The paraffin slices were dewaxed and stained with hematoxylin and eosin (H&E) for morphological analyses. Pulmonary vascular remodeling was quantified by calculating the medial wall thickness. In each group, more than fifty vessels, categorized as 51–150 μm in diameter, were analyzed by an observer unrelated to the experiment. Wall thickness, expressed as a ratio of medial area to cross sectional area (media/CSA), was calculated using ImageJ software (National Institutes of Health, Bethesda, MD, USA).

### 2.6. Generation of EP300 EBS (EGR1 binding site)-KO rats

EP300 EBS-KO rats were generated with CRISPR genome-editing system in the SD rats. Briefly, several pairs of single guide RNAs (sgRNAs) flanking the last two EBS of EP300 promoter were designed using an online CRISPR design tool.^1^ Then, Cas9/sgRNA plasmid construction was completed and the sequences were further confirmed by DNA sequencing. UCA™ (Universal CRISPR Activity Assay), a sgRNA activity detection system developed by Biocytogen, was performed to screen two candidate sgRNAs for the next step. Both pT7-sgRNA and pT7-Cas9 plasmids were constructed and used to prepare sgRNAs and Cas9 mRNA with T7 Ultra Kit (Life Technologies). Purified Cas9 mRNA and sgRNAs were mixed and injected into the cytoplasm of fertilized eggs. PCR analysis was performed to verify the rat genotypes, primers were used as follows, forward: 5′- ATT TCT CCT CCA CGG GCT TG-3′ and reverse: 5′- GGA GGA GGG TAC AGC AAC AC-3′. The wild-type allele yielded an amplicon of 588 bp, whereas the mutated allele yielded an amplicon of 304 bp.

### 2.7. Cell culture and transfection

293A and 293T cells were purchased from the American Type Culture Collection, cultured in Dulbecco’s modified Eagle medium (DMEM, CORNING), and supplemented with 10% fetal bovine serum (FBS, Gibco). HPASMCs purchased from ScienCell (ScienCell, #3110, USA) were cultivated in Smooth Muscle Cell Medium (SMCM, ScienCell, #1101, USA). HPASMCs passaged to 5–7 generations were used for all cell culture experiments. All cells were cultured at 37°C in 5% CO_2_ and 95% relative humidity.

### 2.8. Isolation of rat PASMCs

Primary rat PASMCs (RPASMCs) were isolated from male SD rats (6-week-old) as described (18). The pulmonary artery was isolated under sterile conditions and excess tissue around the blood vessels was removed. The pulmonary artery was cut into small pieces in PBS containing 1 mg/ml collagenase type II (Worthington Biochemical Corp.) and 0.5 mg/ml Elastase type IV (Sigma-Aldrich). It was then digested for 60 min at 37°C. Finally, the isolated single cells were collected by centrifugation (1,000 g for 10 min) and incubated in DMEM supplemented with 10% FBS (Gibco) and 100 U/ml penicillin-streptomycin (HyClone^®^). The purity of the RPASMCs was checked by immunofluorescence staining with antibody against α-smooth muscle actin (α-SMA, Sigma Aldrich).

### 2.9. Gene overexpression or silencing assessment

Gene overexpression in RPASMCs was performed *via* lentivirus-mediated approaches. Chemical synthesized siRNAs (Shanghai GenePharma Co.) were used to silence EP300 expression in primary RPASMCs or HPASMCs using GeneSilencer (Genlantis) as transfection reagent. Two different siRNA sequences targeting rat EP300 were used. Then qRT-PCR was performed 48 h later to analyze the expression level of EP300 in RPASMCs and HPASMCs. The β-actin was used as endogenous control.

### 2.10. Quantitative real-time PCR

RNAs were extracted from cultured cells or tissues using RNAiso Plus (Takara). To detect mRNA, total RNAs were reverse transcribed with M-MLV reverse transcriptase using Oligo d(T_24_) as primer. Relative mRNA expression was determined using SYBR Green Master Mix (Thermo Scientific™) on an ABI StepOne real-time PCR system (Applied Biosystems); β-actin served as an internal control for the normalization of mRNA expression. All the sequences for qPCR primers include β-actin, forward: 5′-AAG TCC CTC ACC TCC CAA AAG-3, reverse: 5′-AAG CAA TGC TGT CAC CTT CCC-3′; EP300, forward: 5′- TCA AGA TCG CTT TGT CTA CAC CT-3, reverse: 5′- TGG CTG CGG CTT GCT GGT TG-3′; EGR1, forward: 5′- GCG CTG GTG GAG ACA AGT TA-3, reverse: 5′- AAG TGT TGC CAC TGT TGG GT-3′.

### 2.11. Western blot

The gene expression on translational level in cells and tissues were evaluated by western blot assay. Briefly, cells were lysed with ice-cold RIPA buffer (50 mM Tris-HCl, pH 7.5, 1 mM EDTA, 150 mM NaCl, 1% NP-40, 0.25% deoxycholate) containing protease inhibitor. After equal amounts of protein were electrophoresed on a sodium dodecyl sulfate-polyacrylamide gel (SDS-PAGE), they were transferred to PVDF membranes (Millipore). Membranes were blocked with TBST buffer (50 mM Tris, 150 mM NaCl, pH7.4, 0.1%Tween-20) containing 3% BSA for 1 h and incubated with primary antibodies overnight at 4°C. The primary antibodies used in this study included: anti-EP300 (Sigma Aldrich, 05-257), anti-EGR1 (Cell Signaling Technology, #4154S) and anti-β tubulin (Proteintech, 10094-1-AP). Membranes were then incubated with horseradish peroxidase-conjugated anti-rabbit (BIO-RAD, 170-6515) or anti-mouse (Jackson ImmunoResearch, 115-005-003) secondary antibodies for 1 h. The protein bands were visualized with a Super Signal chemiluminescent detection module (Thermo Scientific Inc.), and pictures were taken using an automatic chemiluminescence image analysis system (BLT Gel View 6000Plus).

### 2.12. Cell proliferation assay

Cell proliferation was determined by 5′-ethynyl-2′-deoxyuridine (EdU) incorporation. EdU labeling was performed with an EdU Assay Kit (Ribobio, Guangzhou, China) according to the manufacturer’s recommendation. Briefly, ∼1 × 10^4^ cells were seeded in 48-well plates at a density of ∼60%. After overnight culture, cells were transfected with siRNA against EP300 or scramble control. Two days after the transfection, the cells were incubated with 20 mM EdU for 4 h at 37°C, then fixed in 4% paraformaldehyde for 30 min at room temperature and permeabilized in 0.5% Triton X-100 for 10 min. After washing with PBS, the cells were incubated with 200 μl 1 × Apollo reaction cocktail for 30 min. DNA was then stained with 1 mg/ml of Hoechst for 10 min and Images were taken and analyzed with the Lionheart FX Automated Live Cell Imager (BioTek^®^, USA).

### 2.13. Cell apoptotic assay

Annexin V-FITC/PI staining kit (KeyGen Biotechology, Nanjing, China) was used to detect cell apoptosis following the manufacturer’s instruction. Apoptotic cells were measured by using FACS Aria flow cytometry (BD FACSAria™ III, BD Biosciences, Franklin Lakes, NJ, USA) and the data was analyzed using Cell Quest software (BD Biosciences).

### 2.14. ChIP-PCR

BeyoChIP™ Enzymatic Chromatin Immunoprecipitation (ChIP) Assay Kit (KeyGen Biotechology) was used to conduct ChIP experiments for human EP300 promoter in 293T cells with PCR forward primer (5′- ACAAGAGACACTCACCTCCT –3′) and reverse primer (5′- CGAGTTCTCTGCGGCCATTA –3′).

### 2.15. Dual-luciferase assay

Human embryonic kidney (HEK) 293 cells (3 × 105 cells per well in 12-well plate) were co-transfected with 200 ng of the mutation/wild type of the EP300 promoter and the EGR1 overexpression vector (or control vector). Activities of firefly luciferase (FL) and Renilla luciferase (RL) were measured 48 h after transfection.

### 2.16. Statistical analysis

All data shown are mean values with standard deviation (SD) of at least three experiments. When only two groups were compared, the significant difference was assessed with a double-sided Student’s *t*-test. A significant difference between groups was analyzed using one-way ANOVA, and *p*-value less than 0.05 was considered significant.

## 3. Results

### 3.1. EP300 expression is upregulated in MCT-induced PH model in rats

**FIGURE 1:**
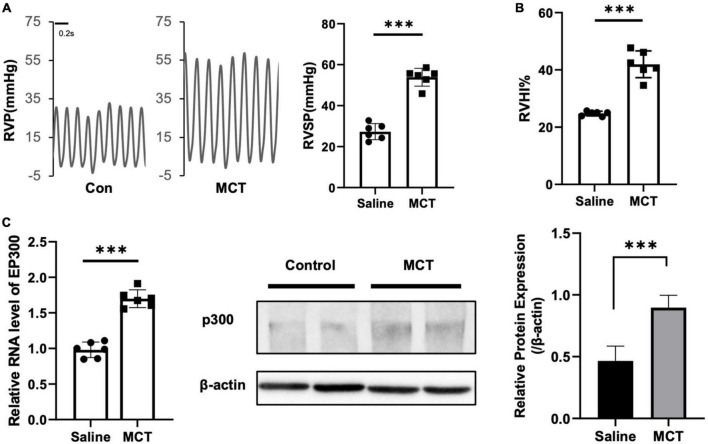
EP300 expression is upregulated in pulmonary arteries of MCT-induced PH rat. Rats (8-wk old, male, WT, 6 rats per group) were injected with saline or monocrotaline (MCT, 50 mg/kg) for 3 weeks. **(A)** The right ventricular systolic pressure (RVSP). **(B)** The right cardiac hypertrophy index [RVHI, RV/(LV + S)]. **(C)** The expression level of EP300 in pulmonary arteries (PAs) from saline or MCT-treated rats was assessed by qRT-PCR and western blot, and β-actin was used as endogenous control. ^***^p < 0.001, compared between indicated groups, student’s *t*-test was used for all the experiments.

It has been reported that several proteins associated with PH pathogenesis are acetylated and modulated by EP300, indicating that EP300 might be involved in PH development. To test this hypothesis, we induced PH in rats using MCT. It was found that the right ventricular systolic pressure (RVSP, Figure 1A) and right ventricle hypertrophy index (RVHI, Figure 1B) significantly increased 3 weeks after MCT administration. The EP300 expression of in pulmonary arteries was found significantly upregulated after MCT treatment on both transcriptional and translational levels (Figure 1C), analyzed by qRT-PCR and western blot assay, implying a tight correlation between EP300 and PH.

### 3.2. EP300 knockdown aggravates MCT-induced PH

**FIGURE 2:**
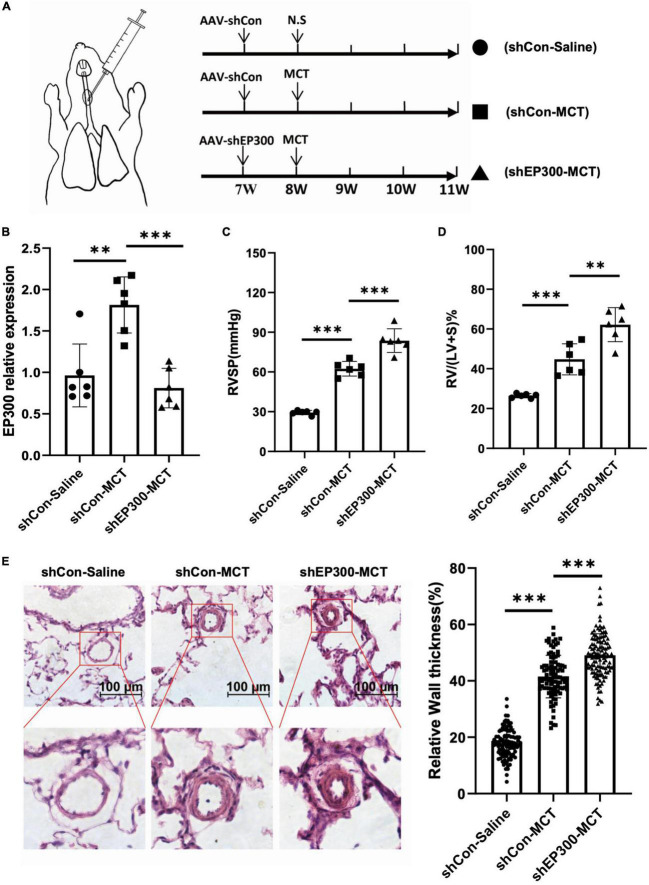
EP300 knockdown in PAs aggravates MCT-induced PH. **(A)** The diagram of establishing MCT-induced PH rats or control rats treated with different of AAV9-shRNA. One week before injecting saline or MCT, AAV-shCon or AAV-shEP300 was intratracheally delivered to the rats (7-wk old, male, 6 rats per group). **(B)** The mRNA expression level of EP300 in the pulmonary arteries from rats (treated with saline or MCT) infected with the AAV virus, assessed by qRT-PCR, and β-actin was used as endogenous control. **(C)** RVSP of the rats in each group. **(D)** RVHI of the rats in each group. **(E)** Left panel: representative pulmonary artery images (H&E staining) in the lung sections of experimental rats (scale bars: 100 μm); right panel: quantification of the relative wall thickness of pulmonary artery in each group [PAs wall thickness = (total perimeter–lumen perimeter)/total perimeter]. *p < 0.05, ^**^p < 0.01, ^***^p < 0.001, compared between indicated groups, student’s *t*-test was used for all the experiments.

To confirm the physiological significance of EP300 upregulation induced by MCT, AAV9 virus expressing shRNA against EP300 or control were injected into the lung of SD rats through the trachea, and the effect of EP300 knockdown on MCT-induced PH was evaluated (Figure 2A). The RNA level of EP300 was significantly increased in pulmonary arteries after MCT treatment, whereas augmenting of EP300 was abrogated by AAV9 expressing shEP300 (Figure 2B). Then the hemodynamics were assessed, and as the results suggest, the elevation of RVSP and RVHI induced by MCT were exacerbated by EP300 knockdown (Figures 2C, D). Small pulmonary arterial remodeling was also investigated *via* H&E staining. Similarly, compared with the rats infected with AAV9-shCon, vascular thickening and lumen closure were exacerbated in the AAV9-shEP300 infected group (Figure 2E). Taken these data together, EP300 knockdown could aggravate MCT-induced PH, indicating the upregulation of EP300 in MCT-induced PH model provided protection from further deterioration.

### 3.3. EP300 repression promotes PASMCs proliferation

**FIGURE 3:**
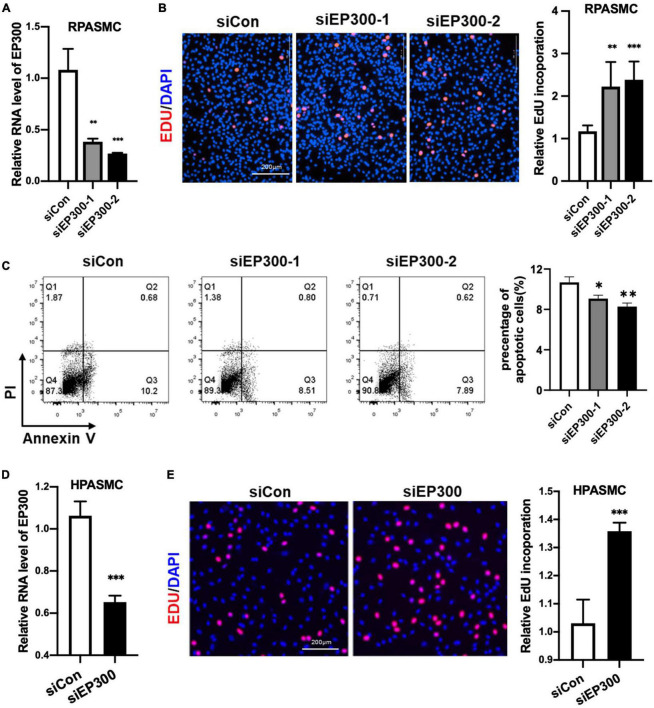
EP300 knockdown promotes the proliferation and inhibits the apoptosis in PASMCs. **(A)** The evaluation of EP300 siRNAs (siEP300-1 and siEP300-2) knockdown efficiency in rat PASMCs (RPASMCs) on the transcriptional level. The RPASMCs were transfected with siRNAs against EP300 (siEP300) or scramble negative control (siCon), and qRT-PCR was performed 48 h later to analyze the expression level of EP300. The β-actin was used as endogenous control. The siEP300-1 and siEP300-2 were different sequences targeting EP300. **(B)** The effect of EP300 knockdown on RPASMCs proliferation. Left panel: EdU assay of RPASMCs transfected with siCon or siEP300 for 48 h. The cells were incubated with EdU solution for 4 h, followed by formalin fixation and staining with Apollo dye (red) and DAPI (blue). Right panel: bar graph of relative EdU incorporation rate in each group. **(C)** The effect of EP300 knockdown on RPASMCs apoptosis. Left panel: flow cytometric analysis of apoptosis; right panel: bar graph of relative apoptosis rate in each group. **(D)** The silencing efficiency of EP300 in human PASMCs (HPASMCs). The cells were transfected with siEP300 or siCon for 48 h, and qRT-PCR was performed to analyze the expression level of EP300. The β-actin was used as endogenous control. **(E)** The effect of EP300 knockdown on HPASMCs proliferation. Left panel: EdU assay of HPASMCs transfected with siCon or siEP300 for 48 h (performed as described above); right panel: bar graph of relative EdU incorporation rate in each group. *N* = 3, bar graphs are mean ± SD. *Represents the significance between the two groups of siCon and siEP300. ^**^p < 0.01, ^***^p < 0.001, compared between siEP300 and siCon, student’s *t*-test was used for all the experiments.

How does EP300 affect PH development? As known, abnormal proliferation of PASMCs is a vital cause of pulmonary arterial remodeling and PH development. Therefore, we explored the effect of EP300 intervention on PASMCs proliferation. Two different siRNAs were used to repress the expression level of EP300 in RPASMCs. Both siEP300-1 and siEP300-2 were verified with significant interfering efficiency by qRT-PCR (Figure 3A). EdU incorporation assay was then performed, and the results indicated that EP300 knockdown significantly accelerated RPASMCs proliferation (Figure 3B). In addition, the apoptosis of RPASMCs were reduced significantly due to the loss of EP300 as determined by flowcytometry with Annexin V/PI staining (Figure 3C). Similarly, EP300 knockdown significantly promoted EdU incorporation in human PASMCs (HPASMCs, Figures 3D, E). Taken together, those data suggest that EP300 repression promotes PASMCs proliferation, involving in pulmonary artery remodeling and PH development.

### 3.4. EP300 is regulated by EGR1 in PASMCs

**FIGURE 4:**
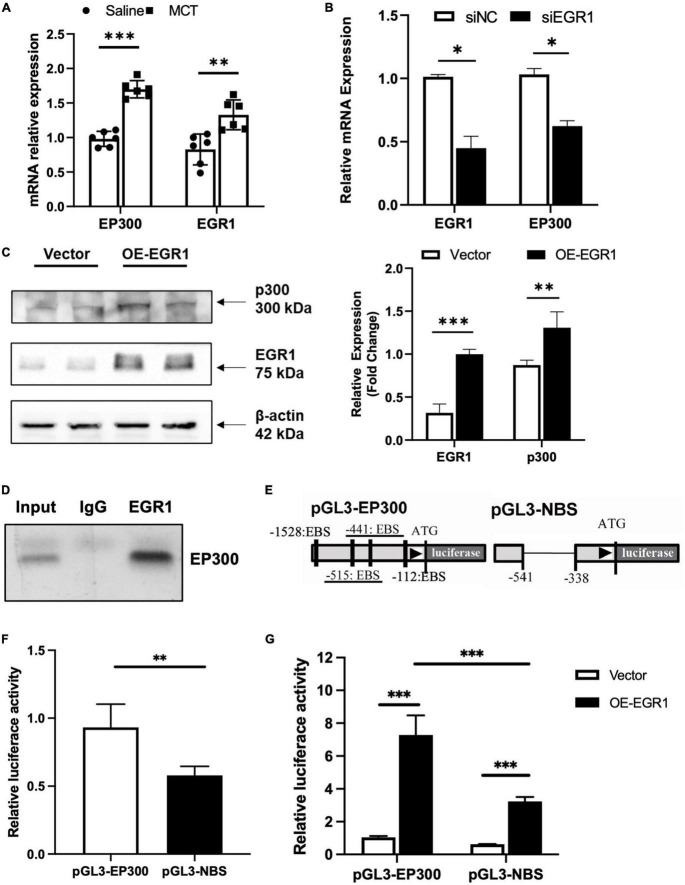
EP300 is regulated by EGR1. **(A)** The relative expression of EGR1 and EP300 in PAs of rats treated with Saline or MCT by qRT-PCR. The β-actin was used as endogenous control. **(B)** The relative expression levels of EGR1 and EP300 determined by qRT-PCR in RPASMCs transfected with siRNA against EGR1 (siEGR1) or scramble control (siCon) for 48 h. The β-actin was used as endogenous control. **(C)** The protein level of EGR1 and p300 in RPASMCs infected with lentivirus overexpressing EGR1 or control without insert for 96 h. Left panel: western blotting picture; right panel: bar graph of relative protein level. The β-tubulin was used as endogenous control. **(D)** Chip-PCR was performed to analyze the binding relationship between EGR1 and EP300 promoter. **(E)** Construction of luciferase reporter vectors containing WT human EP300 promoter (pGL3-EP300) or EP300 promoter lacking two EBS (pGL3-NBS). **(F)** The luciferase assays of pGL3-EP300 or pGL3-NBS vector in transfected 293A cells. EP300 promoter activity was measured by dual luciferase assay. **(G)** The dual luciferase assays of pGL3-EP300 or pGL3-NBS vector co-transfected with the EGR1 overexpression vector (or control vector) 48 h after transfection. *N* = 3, bar graphs are mean ± SD. *Represents the significance between the two groups of siCon and siEP300. ^**^p < 0.01, ^***^p < 0.001, compared between indicated groups, student’s *t*-test was used for all the experiments.

It has been reported that EP300 could be regulated by EGR1 in prostate cells (19). Therefore, we explored whether MCT-induced the increase of EP300 in the pulmonary arteries was dependent on EGR1 regulation. Firstly, the mRNA expression level of EGR1 was examined by qRT-PCR. Similar to EP300, EGR1 was also upregulated in the pulmonary arteries of MCT-induced PH rats, (Figure 4A), implying that EP300 upregulation might be modulated by EGR1. Next, we explored the implication on a cellular level. As shown in Figures 4B, C, inhibition of EGR1 decreases EP300 expression, and overexpression of EGR1 increases the protein expression level of EP300 in RPASMCs. The ChIP experiment proved that EGR1 would indeed bind to the promoter region of EP300 (Figure 4D). The promoter region of EP300 was analyzed, and four EGR1 binding sites (EBS) were discovered (Figure 4E), as reported in previous study (19). A mutation of the EP300 promoter was constructed by deleting two EBS (underlined in Figure 4E), and the transcriptional activity of the mutant promoter was significantly decreased compared to the EP300 wild-type (WT) promoter (Figure 4F). Moreover, overexpression of EGR1 sharply promoted the transcriptional activity of the EP300 WT promoter as well as the mutant promoter with 2 remaining EBS. However, the extent of increase was significantly less in the mutant promoter (Figure 4G). This data indicates that EP300 is regulated by EGR1 at the transcriptional level.

### 3.5. Knockout of EBS in the EP300 promoter aggravates MCT-induced PH

**FIGURE 5:**
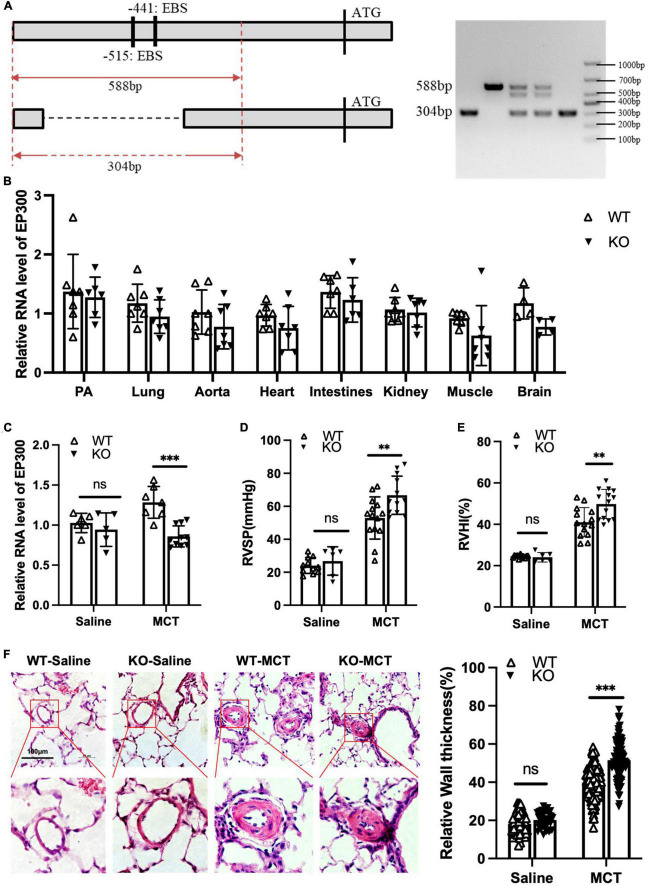
Loss of EBS in EP300 promoter aggravates MCT-induced PH. **(A)** The sketch map of deletion of two EBS in the EP300 promoter region by CRISPR/Cas9 system. Specific primers were designed to confirm the genotype. **(B)** The expression level of EP300 in different tissues of EBS-KO or WT rats analyzed by qRT-PCR and β-actin was used as endogenous control (6 rats per group). **(C)** The expression level of EP300 in pulmonary arteries from EBS-KO or WT rats (treated with saline or MCT) assessed by qRT-PCR and β-actin was used as endogenous control. **(D)** RVSP of EBS-KO or WT rats. **(E)** RVHI EBS-KO or WT rats. **(F)** Left panel: representative pulmonary artery images (H&E staining) in the lung sections of experimental rats (scale bars: 100 μm); right panel: quantification of the relative wall thickness of pulmonary artery of rats in each group. ^**^p < 0.01, ^***^p < 0.001 compared between indicated groups, and “ns” indicates non-significant. Student’s *t*-test was used for all the experiments.

Our data shown above demonstrates that EGR1 promotes EP300 expression in PASMCs. We then explored whether MCT-induced EP300 upregulation is dependent on EGR1 *in vivo*. A knockout (KO) rat model with the deletion of the region containing two EBS in EP300 promoter was constructed using the CRISPR/Cas9 system, and the specific primers designed for genotyping are shown in Figure 5A. The EP300 expression appears to downregulate in most tissues of the KO rat, but not significantly (Figure 5B), indicating that EP300 expression is somewhat independent of EGR1 under normal conditions. However, the MCT-induced EP300 upregulation was almost abrogated in KO rats, suggesting that EGR1 could mediate the increase of EP300 in the MCT-induced PH rats (Figure 5C). Moreover, the PH symptoms, including increased RVSP (Figure 5D), RVHI (Figure 5E), and pulmonary arterial remodeling (Figure 5F) were exacerbated in the KO rats after MCT induction. Hence, these results demonstrate that MCT-induced upregulation of EP300 is dependent on EGR1, which provides a protective effect from PH.

## 4. Discussion

Histone acetylation has been suggested to have a functional role in PH occurrence, and the inhibition of type I and II HDACs has been shown to rescue PH animal models (6, 8, 9). However, whether HATs are involved in this process is not well elucidated. EP300 is one of the most studied HATs participating in the development of many diseases, including cancer and some cardiovascular conditions (10–13, 20). EP300 acetylate histone and many other PAH-related proteins (17), implying a relationship between EP300 and PH. In this study, we aimed to explore the mechanism of this potential relationship. Indeed, EP300 expression was significantly increased in the pulmonary arteries of MCT-induced PH rats (Figure 1C). The MCT-induced PH was aggravated when the EP300 upregulation was abrogated through RNA interference (Figures 3B–E). Our work has shed light on the role of EP300 upregulation in MCT-induced PH, which may be a compensation mechanism to prevent the further deterioration of the disease.

PH is characterized by a marked and sustained elevation of pulmonary arterial pressure and pulmonary vascular resistance. However, the pathological feature of PH is vascular remodeling mediated by pulmonary arterial smooth muscle cell (PASMC) hyperproliferation within the blood vessel wall (21, 22). To explore the cellular role of EP300 in PH development, we investigated the functional role of EP300 in WT PASMCs proliferation and apoptosis. As our results suggest, the knockdown of EP300 accelerates PASMCs proliferation and decreases the apoptosis (Figure 3). However, a recent study reported using RNA to interfere EP300 attenuated the hyperproliferative phenotypes of PAH-SMC and PAH-FBs (14). As we know, PAH-SMC displayed significantly higher proliferation than the normal PASMCs, and EP300 interference could decrease such hyperproliferative but aggravate normal SMC proliferation, suggesting EP300 plays a complex regulatory role in PH occurrence and development.

EP300 is an extensively studied histone acetyltransferase. However, most reports focus on the function of EP300, and little is known about the mechanism of EP300 expression. To explain how the upregulation of EP300 may provide a protective effect during the development of PH, we identified four EGR1 binding sites (EBS) located in the promoter region of EP300, as previously reported (19). It has been reported that EGR1 in pulmonary vascular of PH patients was expressed abundantly (23). And we also found that the expression of EGR1 in the PAs of MCT- induced PH rats was also significantly increased. We hypothesized that EGR1 mediates EP300 upregulation during PH development. Indeed, we found that the inhibition of EGR1 decreased EP300 expression while the overexpression of EGR1 promoted EP300 expression in PASMCs, and the deletion of two EBS affected the transcriptional activity of the EP300 promoter (Figure 2). Moreover, the deletion of these two EBS in the EP300 promoter eliminated the MCT-induced upregulation of EP300 in rats and aggravated PH development (Figure 5). These results suggest that EGR1-mediated upregulation of EP300 has a protective effect on MCT-induced PH. However, further work is needed to explore whether EGR1 overexpression can also relieve PH development since EP300 is only one of its target genes (19).

Since EP300 upregulation could alleviate PH symptoms, we question whether EP300 overexpression is a potential strategy for PH treatment. However, EP300 overexpression can lead to negative impacts on other tissues. For example, EP300 promotes the development of myocyte hypertrophy and represents a pathway that leads to decompensated heart failure (10, 11). Overexpression of EP300 could also promote LV remodeling after myocardial infarction (11). EP300 is a widely expressed transcriptional coactivator and a major lysine acetyltransferase in multicellular animals. It regulates transcription by serving as a scaffold that connects sequence-specific DNA binding factors and the basal transcriptional machinery (24). It also facilitates transcription through histone acetylation, transcription factors, and autoacetylation (25–28). Hence, EP300 is an upstream regulator that affects many biological processes by modulating the expression of numerous genes. Therefore, targeting EP300 may not be the best strategy for disease treatment, including PH. Exploring PH-related targets regulated by EP300 could lead to the discovery of hopeful strategies for PH treatment.

## 5. Conclusion

To summarize, the current work reveals that EP300 expression is increased in the MCT-induced PH model mediated by EGR1, and EP300 has the potential to provide protection from PAH. The downstream targets of EP300 and coregulatory mechanisms involved in the protective effect of EP300 in MCT-PH remain to be investigated in future research work.

## Data availability statement

The raw data supporting the conclusions of this article will be made available by the authors, without undue reservation.

## Ethics statement

This animal study was reviewed and approved by the Animal Care and Use Committee of Shenzhen University.

## Author contributions

LY, YZ, and DG: conceptualization. LY and JW: methodology. LY, TW, BL, JT, and JZ: validation. DG: investigation and writing—review and editing. DG, YZ, and JW: resources. LY and YN: writing—original draft preparation. JT, DG, and JL: writing—review and editing. DG and YZ: project administration. DG, YZ, and LL: funding acquisition. All authors contributed to the article and approved the submitted version.

## Footnotes

1
http://www.sanger.ac.uk/htgt/wge/
