# Supplementary material for: The protective role of EP300 in monocrotaline-induced pulmonary hypertension
Source: Front Cardiovasc Med. 2023 Feb 22;10:1037217. doi: 10.3389/fcvm.2023.1037217 (PMC9992637; doi:10.3389/fcvm.2023.1037217)
Supplement: Supplementary file 1 [file Table_1.DOCX]

Supplementary Material

**The protective role of EP300 in Monocrotaline-induced Pulmonary hypertension**

**Lei YANG^1^ ^†^, Jinglin TIAN^1^ ^†^, Jun WANG^1^, Jie ZENG^2^, Ting WANG^1^, Boya LIN^1^, John LINNEMAN^3^, Li LI^1^, Yanqin NIU^1^, Yunhui ZHANG^2*^, Deming GOU^1*^**

*** Correspondence:**

Deming Gou: [dmgou@szu.edu.cn](mailto:dmgou@szu.edu.cn), Yunhui Zhang: [yunhuizhang3188@126.com](mailto:yunhuizhang3188@126.com)

**Supplementary Figure 1.** The expression of target genes downstream of EP300 in RPASMCs. RT-qPCR was used to detect the gene expression after 48h interference. **p* < 0.05, ***p* < 0.01, compared between siEP300 and siCon, Student’s *t*-test was used for all the experiments.
